# Pressure relief and gas outburst prevention in ultra close coal seams a case study on overlying underlying interactions

**DOI:** 10.1038/s41598-025-02464-3

**Published:** 2025-09-30

**Authors:** Songjiang Sang, Jinguo Lv

**Affiliations:** School of Mechanics and Engineering, Liaoning Technical University, Fuxin, 123000 Liaoning China

**Keywords:** Ultra-close distance coal seam mining, Pressure relief and gas outburst prevention, Numerical simulation, Physical similarity simulation, Mining-induced stress redistribution, On-site monitoring, Mining of overlying protective layer, Deformation of the underlying coal seam, Fracture propagation, Methane gas monitoring, Environmental sciences, Natural hazards

## Abstract

Ultra-close coal seams are adjacent coal seams in a mining area with a spacing of less than 5 m. They have strong interactions during mining. Due to the small distance between layers, mining the upper coal seam creates a pressure relief zone. The lower coal seam enters this zone, which helps release gas pressure and prevent outbursts. This process reduces gas drainage work, supports gob-side entry driving, improves mining efficiency, and promotes sustainable coal resource development. During upper coal seam mining, stress often concentrates in residual coal pillars. Mining-induced stress transfers through rock layers to nearby working faces, changing the stress distribution in the lower coal seam. Roof collapse in the goaf can cause instability, making stress changes more complex. This leads to strong strata pressure and increases mining risks. Determining the pressure relief range of the lower coal seam is key to optimizing gas control and ensuring safe mining. This study focuses on the W_8_ coal seam (W_8_-32030 panel) and its underlying W_9-10_ coal seam in Mine No. 6 of Pingmei Co., with the goal of determining the pressure relief and outburst elimination range. The study employed various methods, including numerical simulation, physical similarity simulation, and field monitoring, covering aspects such as plastic zone monitoring, stress and deformation coefficient monitoring, and gas monitoring. By combining the results from different methods, we obtained the pressure relief and outburst elimination range and performed a comparative analysis of the results from each method. The comprehensive analysis of the advantages of the results from different methods not only improved the accuracy of the study but also provided a reliable basis for a more thorough understanding of the pressure relief and outburst prevention effects in ultra-close coal seams. Finally, by integrating the results from all methods, we determined the pressure relief and outburst elimination range of the underlying coal seam to be 27.5 m. The findings of this study provide scientific guidance for practical mining operations, effectively supporting the rational layout of outburst prevention measures during coal mining and ensuring operational safety, thus holding significant engineering application value.

## Introduction

Coal occupies a dominant position in the energy structure of many resource-based countries, including China, and is a crucial cornerstone of their energy security. While in some countries coal is gradually being phased out due to its perception as a high-pollution energy source, in many resource-based nations, including China, coal remains a major component of the energy structure, ensuring the energy security of these countries^1–3^. Close-distance or ultra-close-distance coal seams are widely distributed worldwide^4^. Due to their unique geological conditions (a close-distance coal seam refers to two coal seams with a normal spacing of less than 10 m that significantly interact during mining), they increase the difficulty of coal extraction to some extent^5–10^. The minimal interlayer spacing between the overlying and underlying coal seams in close-distance coal seams results in a high degree of stress influence on the underlying seam caused by the mining of the overlying seam. This increases the risk of gas outbursts and presents significant safety hazards^11–16^. The mechanism of gas outbursts is complex, thus preventive measures for gas outbursts are of significant importance^17–21^.

Close-distance coal seams with gas outburst risks are widely distributed globally. The presence of gas outburst risks, coupled with the unique condition where the interlayer spacing of many close-distance coal seams is less than 5 m, significantly increases the difficulty of coal seam extraction. Eliminating the risk of gas outbursts requires substantial human and material resources. From the perspective of reducing engineering efforts and improving the mining environment, the stress in the coal and rock beneath the goaf is relieved after protective seam mining. This leads to expansion deformation and the formation of fractures^22,23^, while the overlying coal and rock in the goaf collapse, forming a natural caving arch^24,25^.

These changes induce displacement and variations in stress state, gas content, and gas pressure in the surrounding coal and rock above and below the goaf. Additionally, the formation of numerous fractures greatly enhances coal seam permeability, thereby reducing the risk of coal and gas outbursts^26–30^. This enables efficient mining and tunneling within the pressure-relief zone of the protected seam^31–34^.

However, determining the pressure-relief range of the protected seam is critical for protective seam mining. Accurate measurement of the pressure-relief range is essential for safely and effectively guiding coal mine production.

In the context of ultra-close-distance coal seam mining, the underlying coal seam experiences significant pressure effects due to the mining of the overlying coal seam and the presence of coal pillars^35–41^. Most previous studies on close-distance coal seams have focused on those with interlayer spacings greater than 5 m, while research addressing the unique conditions of interlayer spacings less than 5 m remains limited.

This study explores the pressure relief and gas desensitization range of the underlying coal seam under the special condition of an interlayer spacing less than 5 m. The results provide a foundation for roadway excavation along the goaf and contribute to the effective utilization and conservation of coal resources. The research holds critical importance for the safe and efficient mining of ultra-close-distance coal seam groups under complex geological conditions^42–45^.

## Materials and methods

### Object

The Wu_8_-32030 working face of Pingmei Shenhua Six Mine is located in the eastern part of the mining area. To the west of the Wu_8_-32030 working face is the Wu_8_-32020 working face; to the north is the Wu_8_-32050 working face; to the south is the goaf of the Wu_8_-32010 seam; and to the east is the boundary of the mining area. The Wu_8_-32030 working face primarily extracts the Wu_8_ coal seam, while the underlying Wu_9-10_ coal seam remains unmined. Within a range of 300 m from the entry point, the normal distance between the Wu_8_ coal seam and the Wu_9-10_ coal seam is less than 5 m, categorizing it as an extremely close distance coal seam.

Based on the excavation exposure data and exploration borehole statistics from the Wu_8_-32030 working face, the coal seam structure is complex, characterized by a primary coal structure. The coal seam thickness ranges from 0.2 to 3.7 m, with an average thickness of 3.0 m. The coal seam dip ranges from 0 to 8 degrees, averaging 3 degrees, with the dip angle decreasing from the interior to the exterior of the seam. Within a 1500-m range from the entry point, there is an unstable interburden layer in the coal seam with a thickness of 0.1 to 1.2 m, while the outer 400 m contain no interburden layers. Locally, the coal seam thickness may vary due to structural influences, either thinning or thickening.

**Fig. 1:**
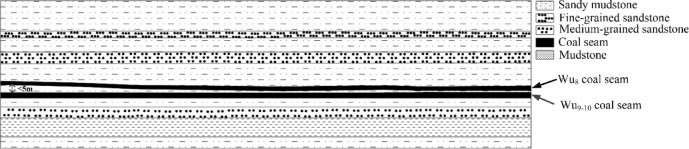
Geological conditions map.

The immediate roof of the Wu_8_-32030 working face consists of sandy mudstone with a thickness of 8 m, while the main roof is medium-grained sandstone, measuring 11.5 m thick. The immediate floor is sandy mudstone with a thickness ranging from 1 to 13 m, with an average thickness of 7 m. The geological conditions are illustrated in Fig. 1.

### Selection of modeling software

In this study, we selected FLAC^3D^ for numerical simulation based on the following considerations compared to Ansys and Salom-meca:

1. Targeted Application and Suitability: Ansys is primarily used for general finite element analysis, while Salom-meca focuses more on mechanical structures and thermodynamic simulations. In contrast, FLAC^3D^ is specifically designed for geotechnical and rock mechanics simulations. It has been widely applied and validated in coal-rock mechanics, stress distribution, and fracture evolution studies.
2. Computational Efficiency and Experimental Validation: FLAC^3D^ offers higher computational efficiency in handling rock strata deformation and can more accurately reflect the mechanical responses in actual mining processes.

Based on these reasons, we ultimately chose FLAC^3D^ for modeling to ensure the rationality and reliability of the simulation results.

### Selection of similar physical simulation test materials and monitoring equipment

#### Selection of similar physical simulation test materials

The test materials mainly consist of two components: aggregates and cementing agents. Aggregates make up a large proportion of the model materials, and their physical and mechanical properties significantly influence the characteristics of the similar materials. In this study, fine sand was selected as the aggregate due to its uniform particle size, good filling capability, and plasticity, which help improve the homogeneity of the model materials. Additionally, the stable mechanical properties of fine sand enable a more accurate simulation of stress transmission characteristics in similar materials.

Lime and gypsum were chosen as the cementing agents. These materials possess excellent bonding properties, effectively enhancing the overall strength and stability of the model materials. Moreover, their high adjustability allows for tuning the mechanical properties by varying the mixing ratio, ensuring that the materials meet the similarity requirements for the experiment.

#### Selection of monitoring equipment for similar physical simulation tests

1. Stress monitoring equipment

**Fig. 2:**
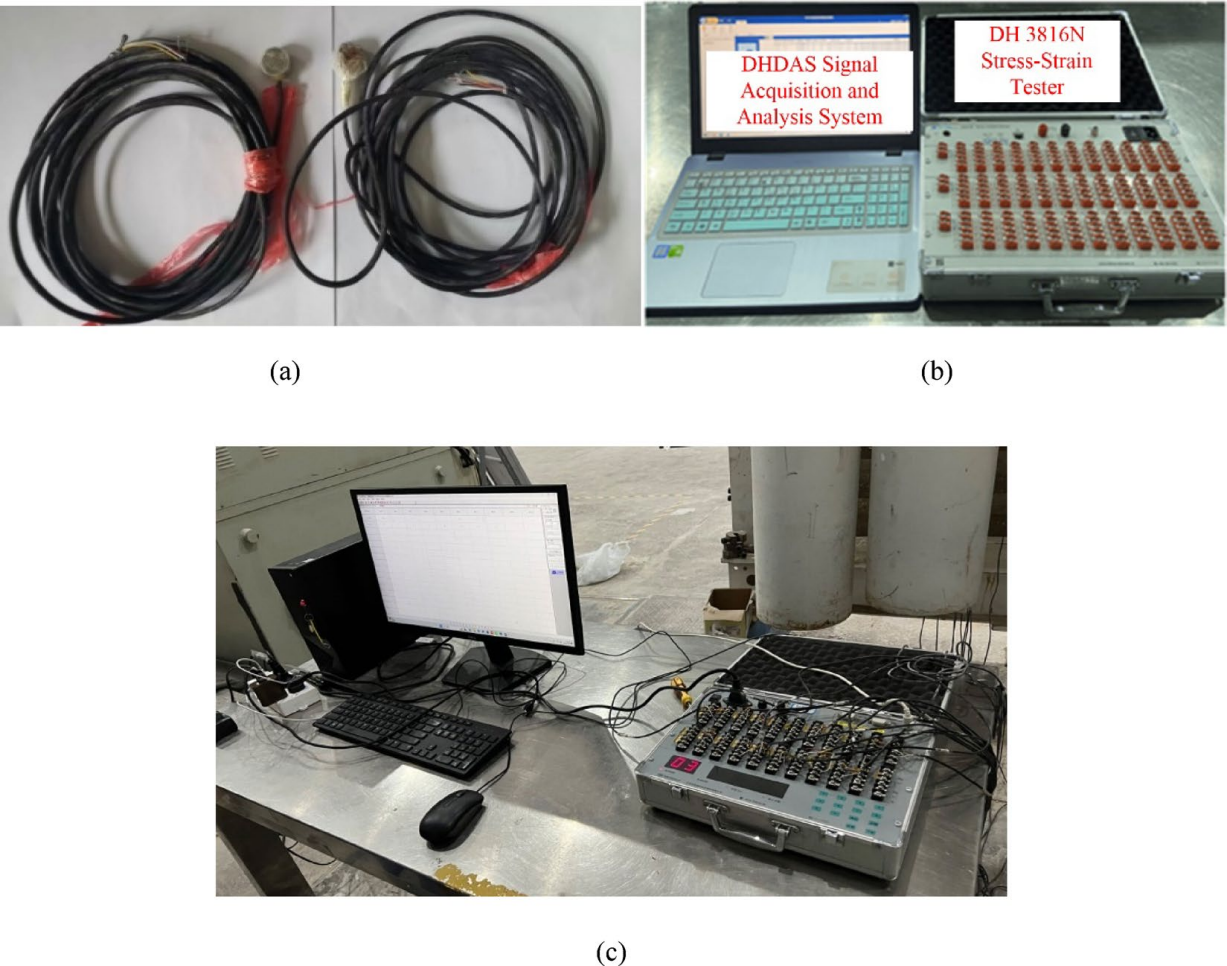
Stress monitoring system. (**a**) LY-350 micro pressure box. (**b**) Static strain gauge. (**c**) Strain acquisition instrument.

In this experiment, the LY-350 miniature pressure box and DH3816N static strain gauge were selected for stress monitoring. Data visualization and analysis were performed using the DHDAS signal acquisition and analysis system on a computer. The pressure box and static strain gauge are shown in Fig. 2.

(2) Deformation coefficient monitoring equipment

**Fig. 3:**
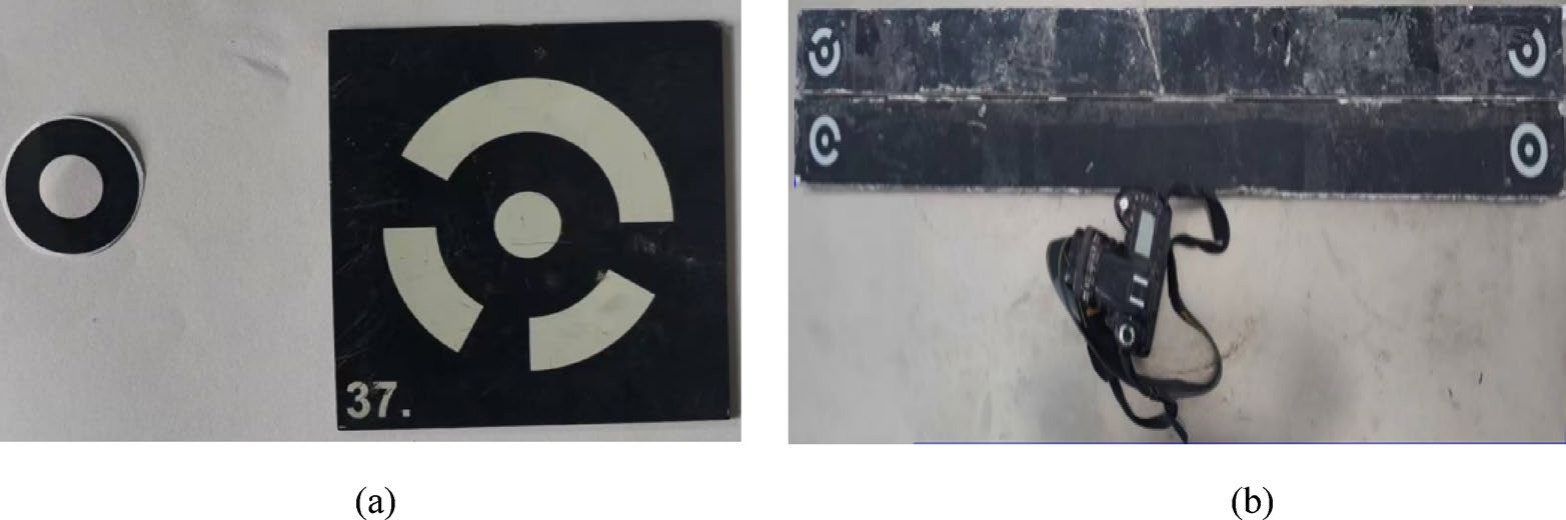
Deformation monitoring based on digital speckle. (**a**) Non-coding point and coding point. (**b**) 3D photogrammetry.

In this experiment, the XJTUDP 3D optical photogrammetry system was used for displacement monitoring of the model. The XJTUDP system includes coded points, non-coded points, and cameras, as shown in Fig. 3.

## Numerical simulation of stress distribution and plastic zone distribution in an ultra-close coal seam model

### Geometric models and physical parameters of coal seams

Based on the occurrence characteristics of key strata in the primary research area of Pingmei No. 6 Mine, the parameters required for the model were determined (see Table 1). The model was appropriately simplified according to the upcoming physical similarity simulation to better align with the similar physical model.

**Table 1:** Physical and mechanical parameters of coal rock layers.

Lithology	Tensile strength (MPa)	Internal friction angle (°)	Elastic modulus (GPa)	Cohesive force (MPa)	Density (Kg·m^−3^)
Fine-grained sandstone	1.00	35.00	4.01	2.00	2950
Sandy mudstone	4.39	39.51	4.16	8.24	2540
Medium-grained sandstone	1.20	37.00	5.99	4.00	2670
Sandy mudstone	4.39	39.51	4.16	8.24	2540
Fine-grained sandstone	1.00	35.00	4.01	2.00	2950
Sandy mudstone	4.39	39.51	4.16	8.24	2540
Medium-grained sandstone	1.20	37.00	5.99	4.00	2670
Sandy mudstone	4.39	39.51	4.16	8.24	2540
Wu_8_ coal seam	0.74	30.20	0.90	6.62	1430
Sandy mudstone	4.39	39.51	4.16	8.24	2540
Wu_9-10_ coal seam	0.80	35.76	1.01	5.00	1460
Sandy mudstone	4.39	39.51	4.16	8.24	2540
Medium-grained sandstone	1.20	37.00	5.99	4.00	2670
Mudstone	0.61	30.00	8.75	1.20	2630
Sandy mudstone	4.39	39.51	4.16	8.24	2540
Medium-grained sandstone	1.20	37.00	5.99	4.00	2670

The FLAC^3D^ software was used to perform creep analysis on the model, with the weak interlayer analyzed using the Burgers-Mohr model. The parameters were determined through field investigations and trial calculations, with the following values: *E*_*K*_ = 1.5 × 10^8^ Pa; *E*_*M*_ = 1 × 10^8^ Pa; *η*_*K*_ = 18.49 × 10^8^ Pa·d; *η*_*M*_ = 6.15 × 10^10^ Pa·d.

**Fig. 4:**
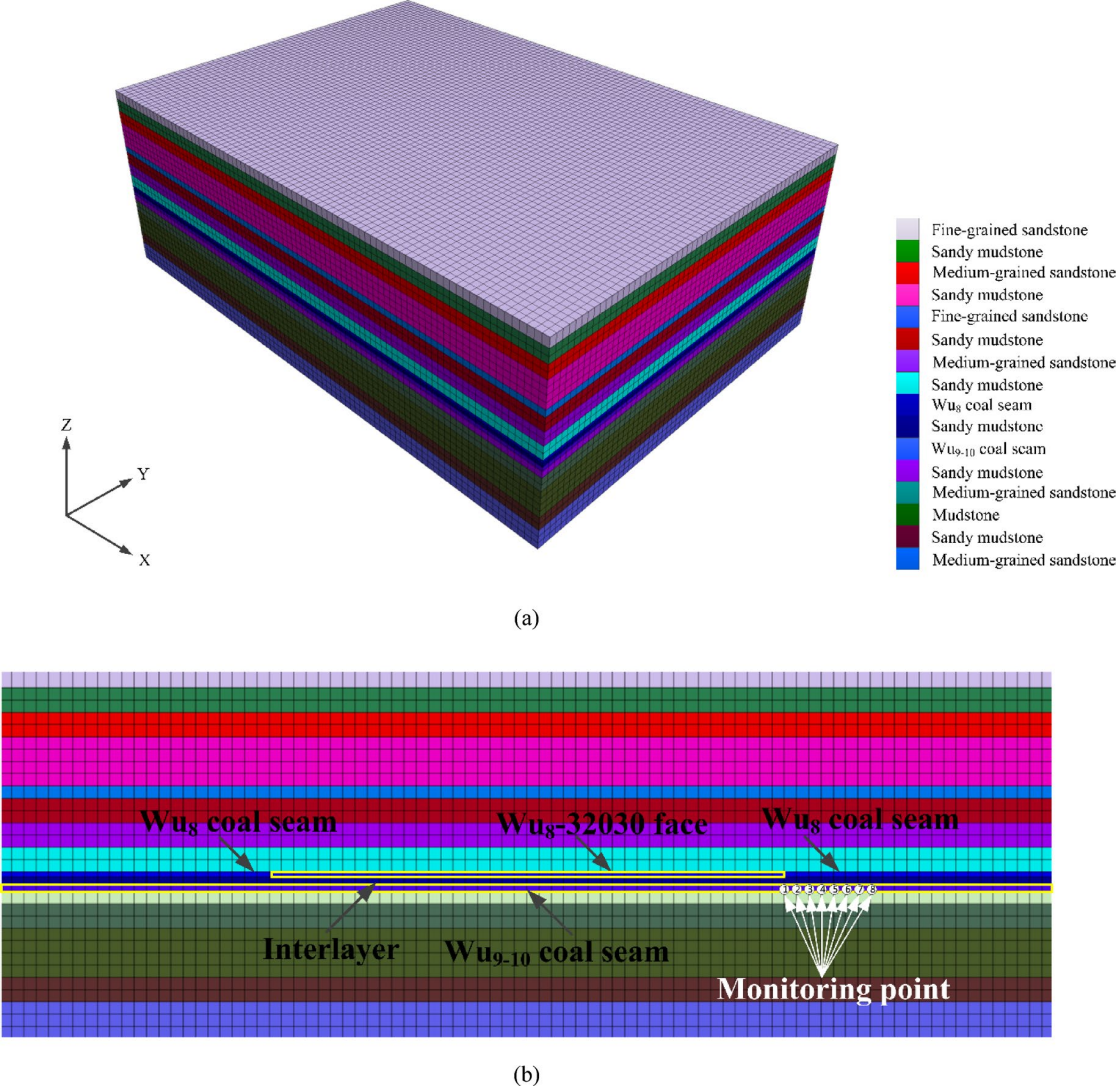
3D Numerical model in FLAC^3D^. (**a**) Three-dimensional numerical model. (**b**) Key monitoring area.

Based on the geological conditions of the Wu_8_-32030 working face and the Wu_9-10_ coal seam, a three-dimensional numerical model was established using the FLAC^3D^ numerical simulation platform. The density of the elements was adjusted according to the importance of the study, and hexahedral elements were used for meshing. The model dimensions were 430 m × 150 m × 300 m, comprising a total of 775,000 elements. See Fig. 4 for details.

The model adopts the Mohr–Coulomb yield criterion, while the Wu_8_ and Wu_9-10_ coal seams utilize the Burgers-Mohr creep constitutive model to simulate the temporal changes in the plastic failure of the coal seams. The boundary conditions of the model are defined as follows: (1) horizontal displacement constraints are applied to the boundaries on all sides of the model; (2) directional displacement constraints are applied to the bottom boundary of the model; (3) a vertical downward load is applied to the top of the model to simulate the overburden weight, with a load of 20 MPa.

### Stress variation characteristics

**Fig. 5:**
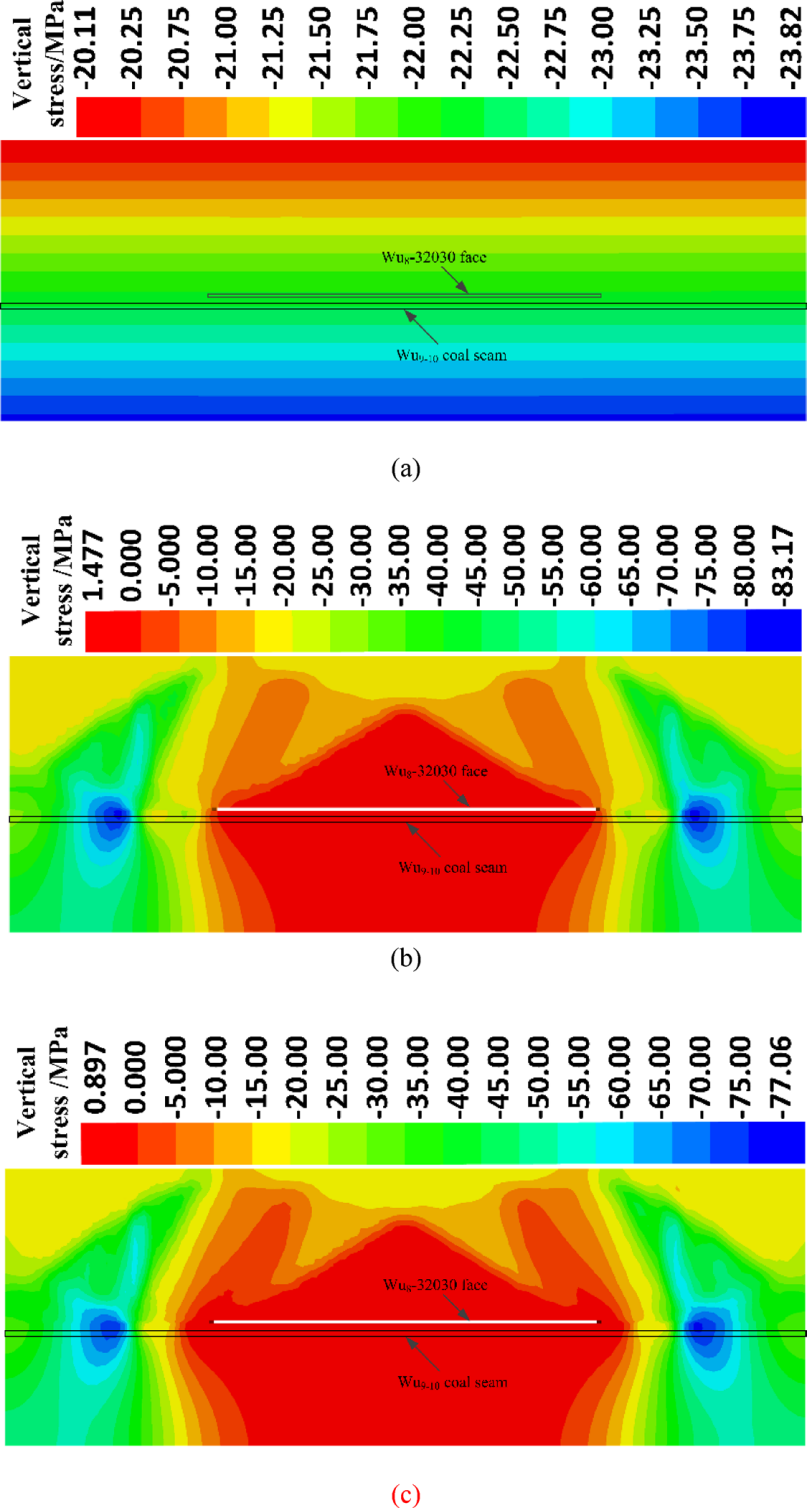

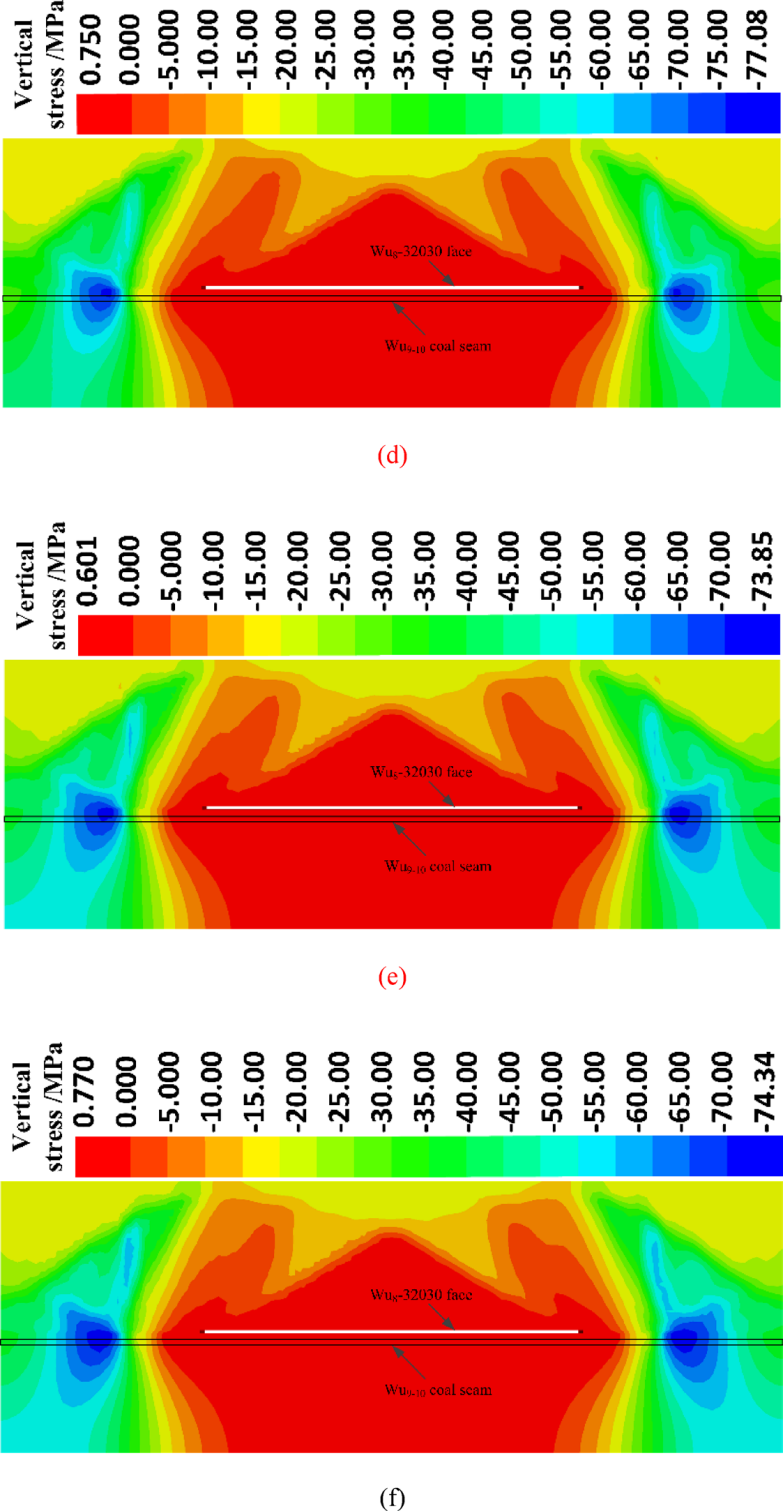
Characteristics of vertical stress distribution. (**a**) Vertical stress diagram before excavation. (**b**) Vertical stress diagram after the face is just excavated. (**c**) Vertical stress diagram 1 month after the face excavation. (**d**) Vertical stress diagram 2 month after the face excavation. (**e**) Vertical stress diagram 3 month after the face excavation. (**f**) Vertical stress diagram 4 month after the face excavation.

The model under initial equilibrium with self-weight and the model immediately after the excavation of the working face adopt the Mohr–Coulomb model. The models for one month, two months, three months, and four months after the excavation of the working face adopt the Burgers-Mohr creep model. The vertical stress calculated by the simulation is shown in Fig. 5.

In the unmined state, after equilibrium is reached due to self-weight, the vertical stress increases regularly with depth, with the vertical stress at the location of the underlying coal seam monitoring points being 21.5 MPa. After the excavation of the working face, a pressure relief phenomenon occurs in the coal seam near the gob area, but the relief range is relatively small. One month after the excavation, the pressure relief range continues to expand. Two months after the excavation, the relief range further increases. After three months, the stress changes little compared to the two-month post-excavation state, indicating that the vertical stress gradually stabilizes and the pressure relief range remains largely unchanged.

### Plastic failure characteristics

**Fig. 6:**
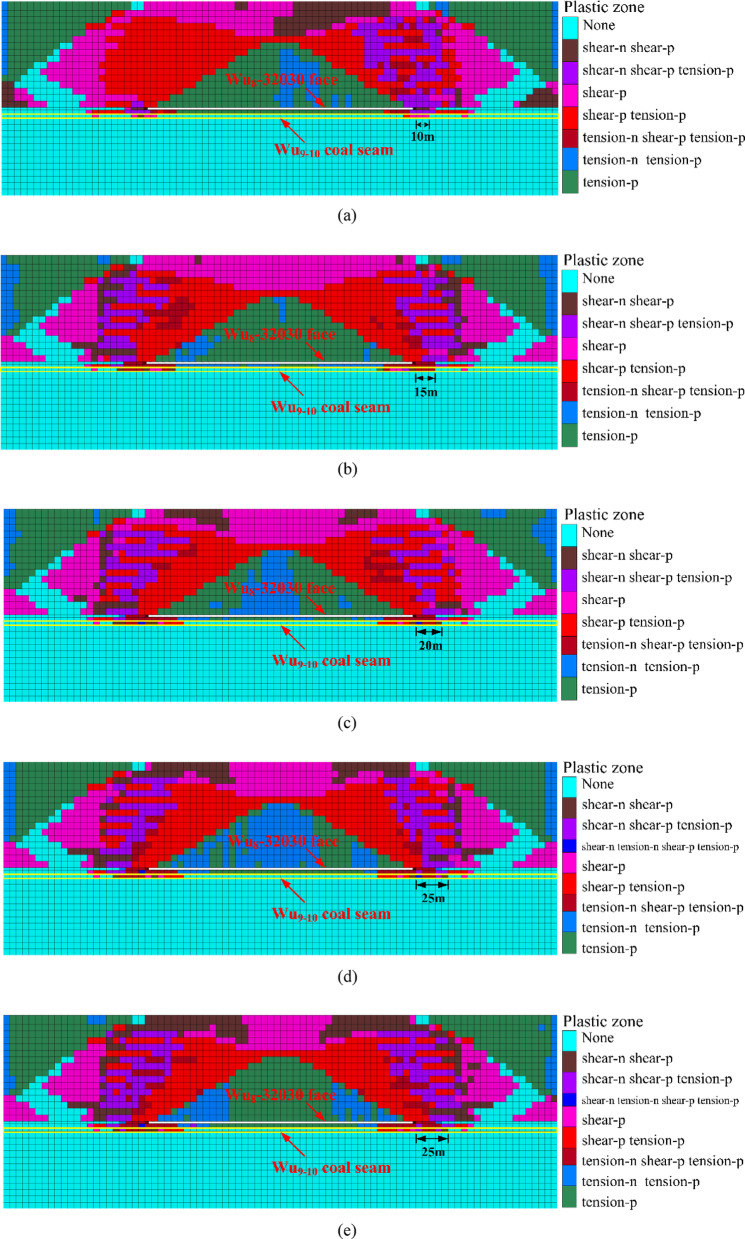
Plastic failure zone expansion cloud map. (**a**) Plastic zone map immediately after face excavation. (**b**) Plastic zone map one month after face excavation. (**c**) Plastic zone map two months after face excavation. (**d**) Plastic zone map three months after face excavation. (**e**) Plastic zone map four months after face excavation.

Figure 6 shows the plastic zone contour maps for the following stages: immediately after the excavation of the working face, one month after excavation, two months after excavation, three months after excavation, and four months after excavation.

Immediately after the excavation of the working face, the plastic zone in the underlying coal seam reaches a range of 10 m. One month after excavation, the plastic zone in the underlying coal seam expands to 15 m. Two months after excavation, the plastic zone further extends to 20 m. Three months after excavation, the plastic zone in the underlying coal seam expands to 25 m. Four months after excavation, the plastic zone remains at 25 m, the same as three months after excavation, indicating that the plastic zone has reached a stable state after three months of excavation.

In conclusion, it can be initially determined that the desorption and pressure relief influence range in the underlying coal seam is approximately 25 m. This will provide a basis for defining the key research area in the upcoming physical similarity simulation.

Due to the consideration of long-term creep effects in the numerical simulation, the stress relief range in the numerical simulation is larger than that in the physical similarity simulation.

## Physical similarity simulation

### Determination of similarity constants and material ratios

**Fig. 7:**
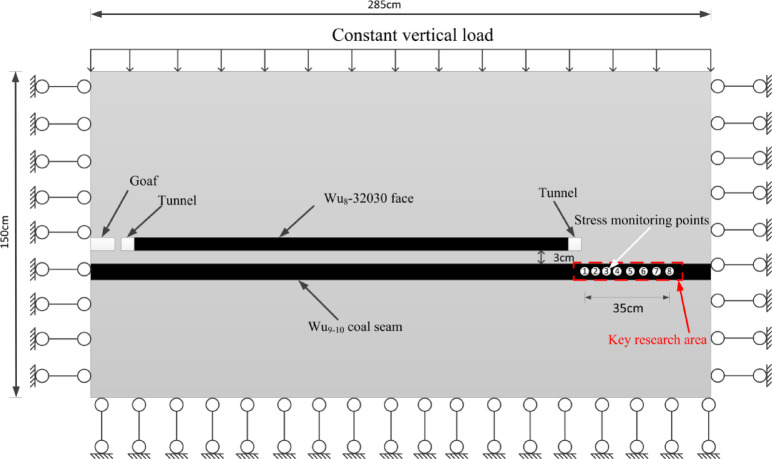
Schematic of measurement point layout.

Based on the characteristics of the working face at the construction site and the occurrence of coal and rock, a physical similarity model was established, and stress monitoring points were installed, as shown in Fig. 7. Utilizing digital speckle technology and stress monitoring techniques, the study examined the crack propagation, stress variation, and deformation coefficient change in the underlying coal seam during the working face mining process. This approach aimed to reveal the depressurization effect of the overlying coal seam mining on the ultra-close underlying coal seam under the influence of mining activities.

Based on actual geological data, the components of the similar simulation materials were selected. The similar simulation materials mainly consist of aggregate and cementing material. The aggregate, which constitutes the majority of the material, serves as the object for cementing by the binder. Its physical and mechanical properties significantly influence the properties of the similar materials. In this experiment, fine sand was selected as the aggregate, while lime and gypsum were used as the cementing materials. A distilled water solution containing an appropriate amount of sodium citrate as a retarder was added. After uniform mixing, the materials were filled and modeled according to the design dimensions. The coal and rock materials were proportioned based on their uniaxial compressive strength.

Referring to the physical and mechanical parameters of the working face and coal-rock strata at the construction site, materials for each rock layer in the model were configured accordingly. The material ratios for the experimental model’s similar materials are shown in Table 2, with a material loss coefficient of 1.05 considered during preparation. Based on the laboratory conditions, the primary parameters considered in this similar simulation experiment include geometric dimensions (*I*), bulk density (γ), displacement (*δ*), strain (*ε*), stress (*σ*), Poisson’s ratio (*u*), elastic modulus (*E*), compressive strength (*P*), and apparent density (*r*).

**Table 2:** Mixing proportion of coal and rock layers.

Num-ber	Lithology	Density (g/cm^3^)	Mixing ratio code	Thickness (m)	Similar thickness (cm)	Apparent density (g/cm^3)^	Fine sand (kg)	Lime (kg)	Gypsum (kg)	Water (kg)
1	Sandy Mudstone	2540	573	16	16	1.5	22.42	3.14	1.35	2.99
2	Fine-grained Sandstone	2950	337	6	6	1.5	20.20	2.02	4.68	2.99
3	Sandy Mudstone	2540	573	8	8	1.5	22.42	3.14	1.35	2.99
4	Medium-grained Sandstone	2670	437	7	7	1.5	21.52	1.61	3.77	2.99
5	Sandy Mudstone	2540	573	17	17	1.5	22.42	3.14	1.35	2.99
6	Fine-grained Sandstone	2950	337	4	4	1.5	20.20	2.02	4.68	2.99
7	Sandy Mudstone	2540	573	8	8	1.5	22.42	3.14	1.35	2.99
8	Medium-grained Sandstone	2670	437	7	7	1.5	21.52	1.61	3.77	2.99
9	Sandy Mudstone	2540	573	9	9	1.5	22.42	3.14	1.35	2.99
10	Wu_8_ coal seam	1430	673	2	2	1.5	23.06	2.69	1.15	2.99
11	Sandy Mudstone	2540	573	3	3	1.5	22.42	3.14	1.35	2.99
12	Wu_9-10_ coal seam	1430	673	3	3	1.5	23.06	2.69	1.15	2.99
13	Sandy Mudstone	2540	573	5	5	1.5	22.42	3.14	1.35	2.99
14	Medium-grained Sandstone	2670	437	7	7	1.5	21.52	1.61	3.77	2.99
15	Mudstone	2630	573	11	11	1.5	22.42	3.14	1.35	2.99
16	Sandy Mudstone	2540	573	7	7	1.5	22.42	3.14	1.35	2.99
17	Medium-grained Sandstone	2670	437	7	7	1.5	21.52	1.61	3.77	2.99
18	Sandy Mudstone	2540	573	5	5	1.5	22.44	3.14	1.35	2.99
19	Mudstone	2630	573	11	11	1.5	22.44	3.14	1.35	2.99
20	Sandy Mudstone	2540	573	7	7	1.5	22.44	3.14	1.35	2.99

Using *t* to represent prototype physical quantities, *m* for model physical quantities, and *C* for similarity ratios, the similarity ratios among the physical quantities can be defined according to the similarity theorem^46^ as follows:

1. Geometric Similarity Ratio $C_{I} = {{I_{t} } \mathord{\left/ {\vphantom {{I_{t} } {I_{m} }}} \right. \kern-0pt} {I_{m} }}$
2. Bulk Density Similarity Ratio $C_{\gamma } = {{\gamma_{t} } \mathord{\left/ {\vphantom {{\gamma_{t} } {\gamma_{m} }}} \right. \kern-0pt} {\gamma_{m} }}$
3. Displacement Similarity Ratio $C_{\delta } = {{\delta_{t} } \mathord{\left/ {\vphantom {{\delta_{t} } {\delta_{m} }}} \right. \kern-0pt} {\delta_{m} }}$
4. Strain Similarity Ratio $C_{\varepsilon } = {{\varepsilon_{t} } \mathord{\left/ {\vphantom {{\varepsilon_{t} } {\varepsilon_{m} }}} \right. \kern-0pt} {\varepsilon_{m} }}$
5. Stress Similarity Ratio $C_{\sigma } = {{\sigma_{t} } \mathord{\left/ {\vphantom {{\sigma_{t} } {\sigma_{m} }}} \right. \kern-0pt} {\sigma_{m} }}$
6. Poisson’s Ratio Similarity Ratio $C_{u} = {{u_{t} } \mathord{\left/ {\vphantom {{u_{t} } {u_{m} }}} \right. \kern-0pt} {u_{m} }}$
7. Elastic Modulus Similarity Ratio $C_{E} = {{E_{t} } \mathord{\left/ {\vphantom {{E_{t} } {E_{m} }}} \right. \kern-0pt} {E_{m} }}$
8. Rock Strength Similarity Ratio ${{P_{t} } \mathord{\left/ {\vphantom {{P_{t} } {P_{m} }}} \right. \kern-0pt} {P_{m} }} = C_{I} {{r_{t} } \mathord{\left/ {\vphantom {{r_{t} } {r_{m} }}} \right. \kern-0pt} {r_{m} }}$

The basic similarity criteria that must be satisfied for analogous simulation experiments can be derived using the equations of elastic mechanics or dimensional analysis, and they are as follows: $C_{\sigma } = C_{I} C_{\gamma }$; $C_{\delta } = C_{I}$; $C_{\varepsilon } = C_{u} = 1$. The dimensions of the model are length × width × height = 285 cm × 30 cm × 150 cm. The model’s sides are fixed to serve as lateral boundary conditions, constraining displacement in the horizontal direction, while the bottom restricts both horizontal and vertical displacements. Considering the existing model size conditions in the laboratory, the geometric similarity ratio for this model is set as $C_{I} = I_{t} /I_{m} =$ 100. At the same time, to prevent difficulties in compacting the model material during mold assembly, a smaller value within a reasonable range of the similarity ratio for bulk density is chosen, with $C_{\gamma } = \gamma_{t} /\gamma_{m} =$ 1.5. Based on the above relationships, the following similarity ratios can be obtained: Displacement similarity constant $C_{\delta } =$ 100, Poisson’s ratio and strain similarity constants $C_{\varepsilon } = C_{u} = 1$, Stress and elastic modulus similarity constant $C_{\sigma } = C_{I} C_{\gamma } = C_{E} = 100 \times 1.5 = 150$.

**Fig. 8:**
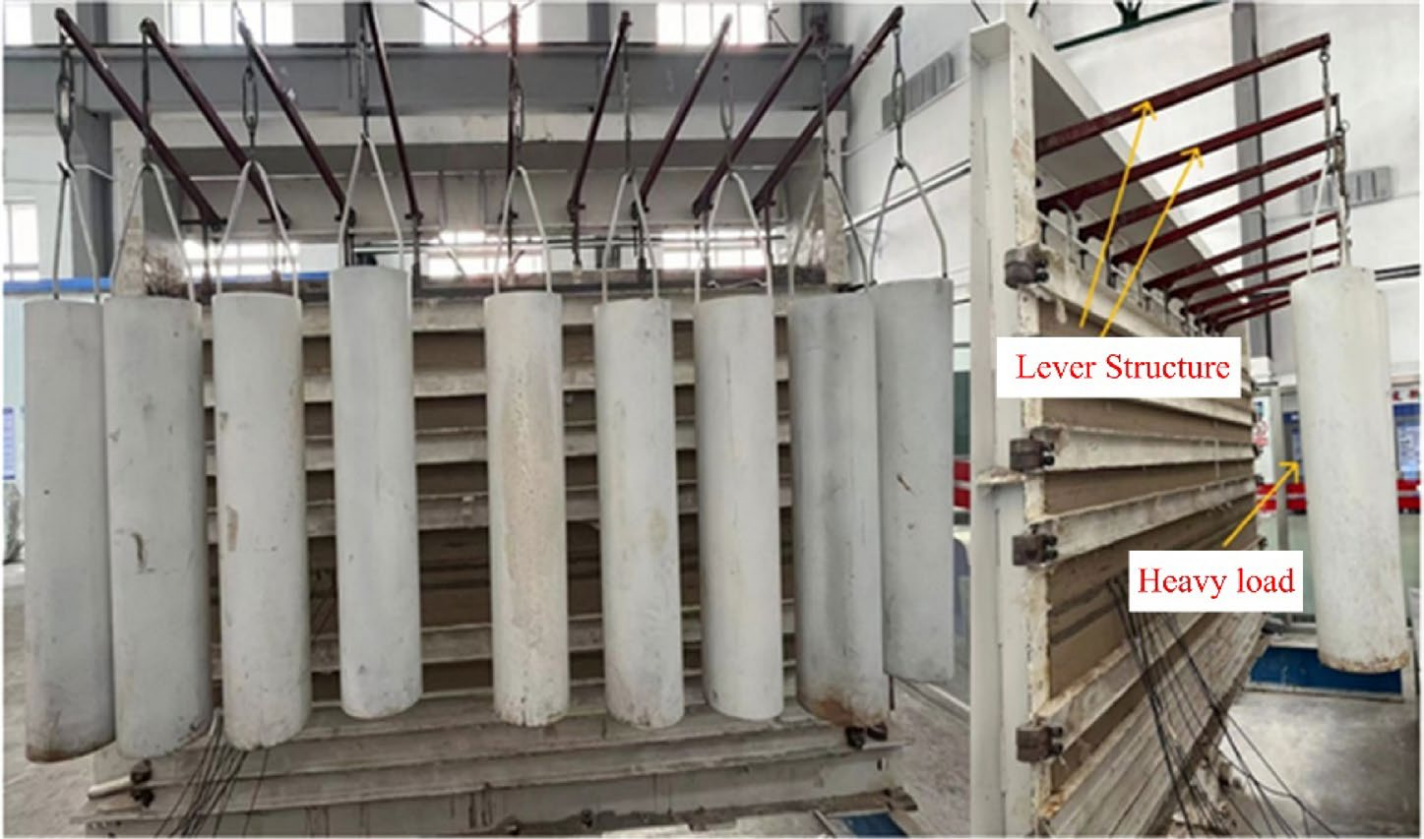
Lever loading system.

**Fig. 9:**
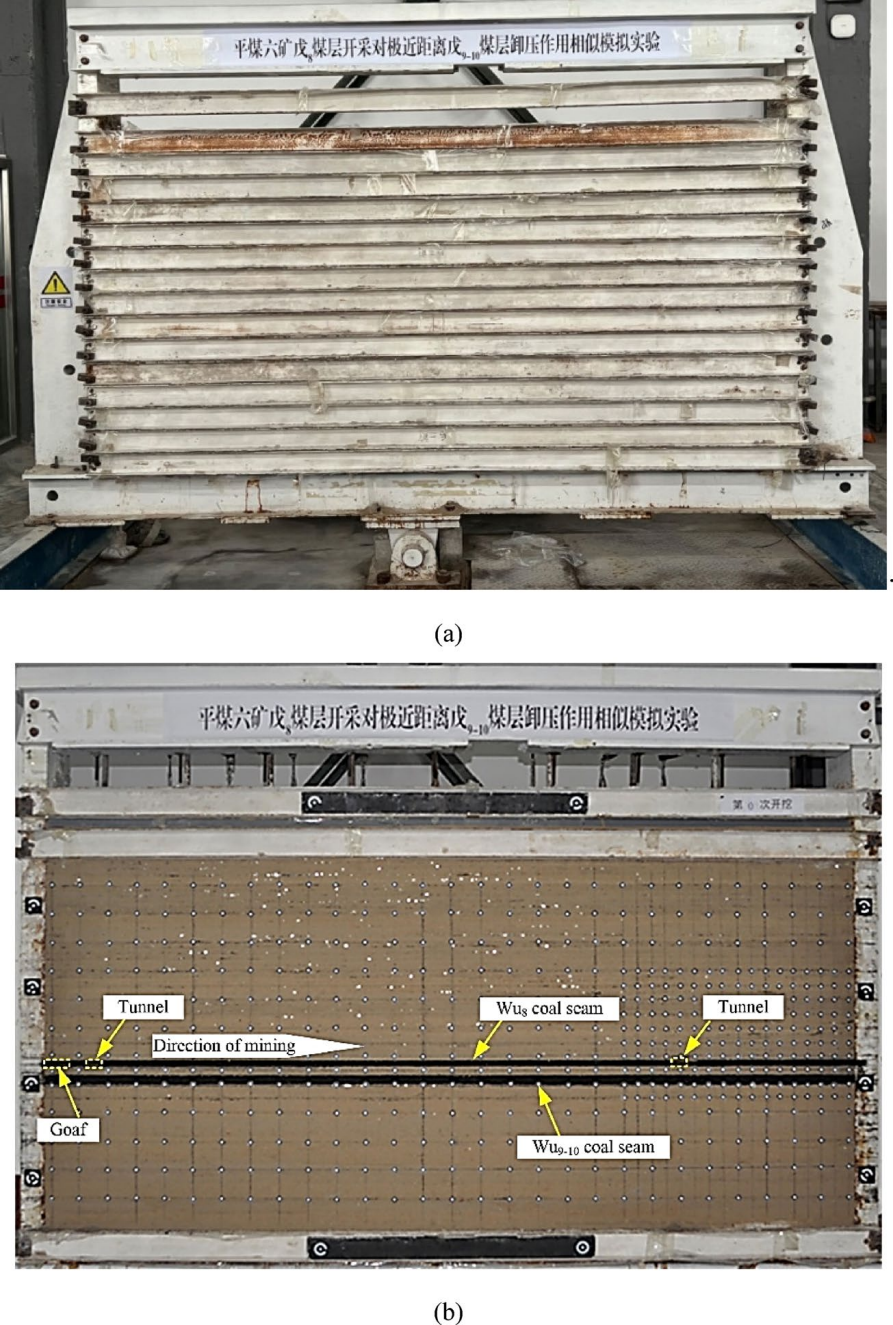
Establishment of the physical similarity model. (**a**) Similarity model maintenance. (**b**) Completion of similarity model construction.

Based on the on-site geological data, the coal seam at the working face is buried at a depth of 899 m, which is relatively deep, and the underlying coal seam reaches a depth of over 900 m. Due to the limited height of the similarity simulation model, it is not possible to proportionally stack the full thickness of the geological layers from the prototype. Therefore, external force compensation is required to make up for the missing self-weight stress. A certain load is applied at the top of the experimental model to simulate the self-weight stress of the overlying rock layers. The overlying load in the model is implemented using external physical weights and lever principles, as shown in Fig. 8. The overlying load is calculated based on the lithology data of the buried strata, and the corresponding value for the similarity simulation model’s overlying load is determined by converting it through the stress similarity ratio. The completed physical similarity model is shown in Fig. 9.

1 $$\sigma_{z} = \sum\nolimits_{i,j = 1}^{n} {\gamma_{i} h_{j} }$$

In the equation: γ_i_ represents the bulk density of each coal and rock layer, in N/m^3^; *i* (Where *i* = 1,2,3…) denotes the index for each coal and rock layer; *h*_j_ is the thickness of each coal and rock layer, in meters; and *σ*_*z*_ is the actual stress value at the location of the coal and rock layer, in Pascals (Pa).

2 $$\sigma_{m} = \frac{{\sigma_{z} }}{{\alpha_{y} }}$$

In the equation: *σ*_*m*_ represents the stress value applied to the model, in Pascals (Pa); α_y_ is the stress similarity ratio. Through calculations, the required overlying load to be applied to the similarity model is found to be 0.119 MPa.

After the construction of the model was completed, the model was cured, with the curing period determined based on temperature, air humidity, and other factors. The curing period for this physical similarity model was set to 12 days. On the 8th day after construction, the mold was removed. After demolding, water was sprayed on the surface of the model every 8 h using a watering can to prevent the surface from becoming overly dry and cracking.

### Characteristics of overburden fracture development

Before excavation, the physical similarity model exhibits no noticeable fractures, and the stress remains in a stable state, as shown in Fig. 9.

**Fig. 10:**
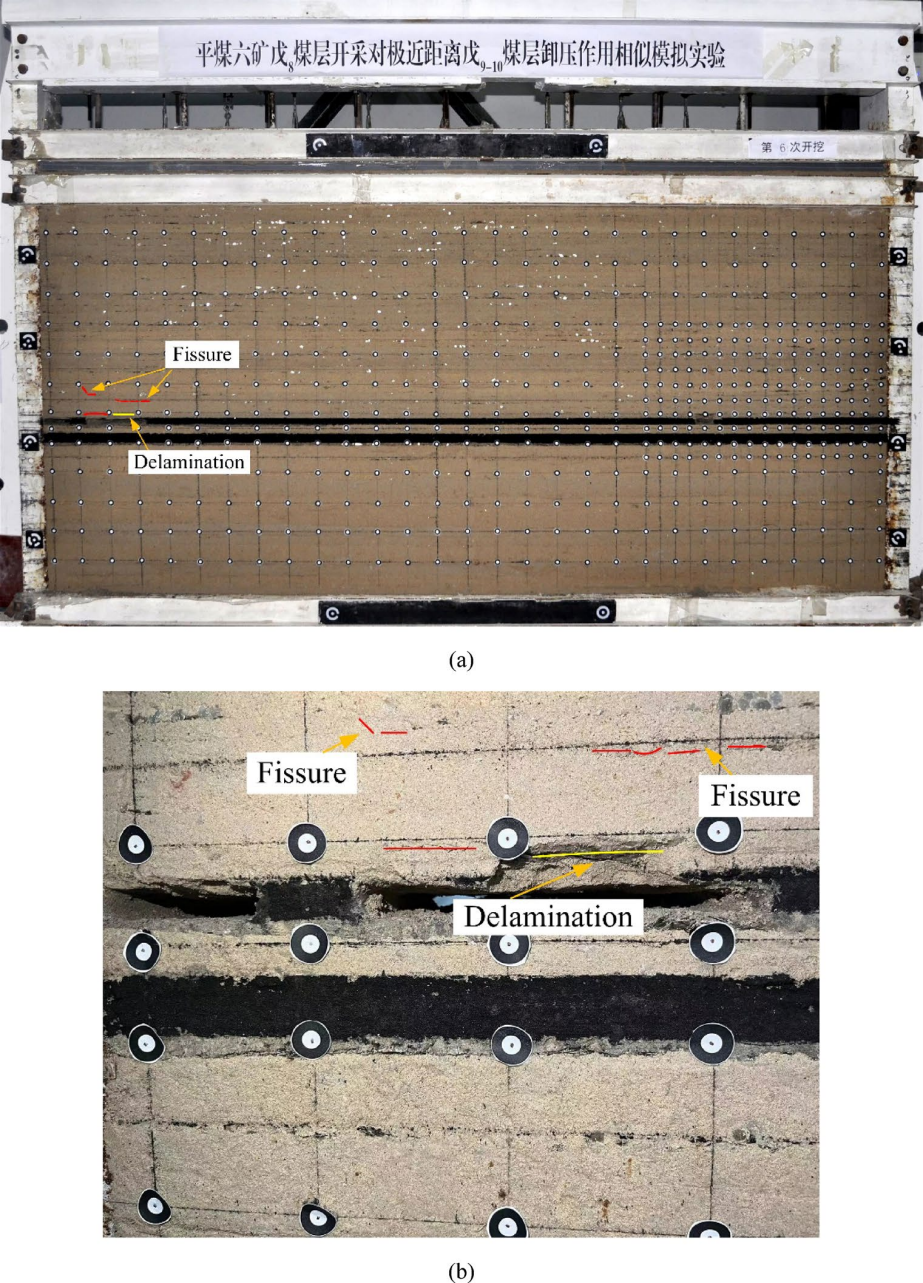
Mining advancement of 18 m. (**a**) Overall deformation and failure. (**b**) Development of local cracks.

**Fig. 11:**
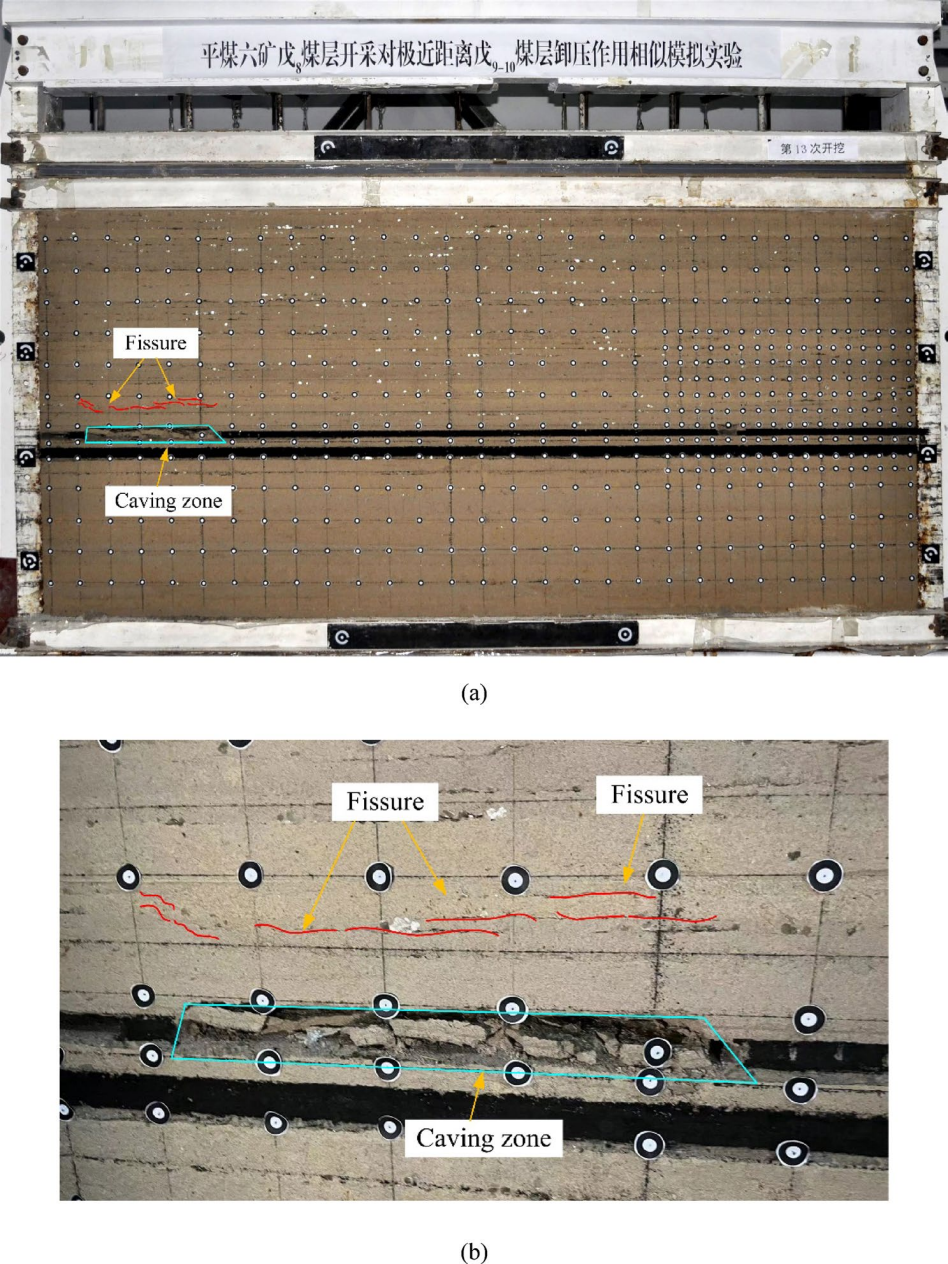
Mining advancement of 39 m. (**a**) Overall deformation and failure. (**b**) Development of local cracks.

**Fig. 12:**
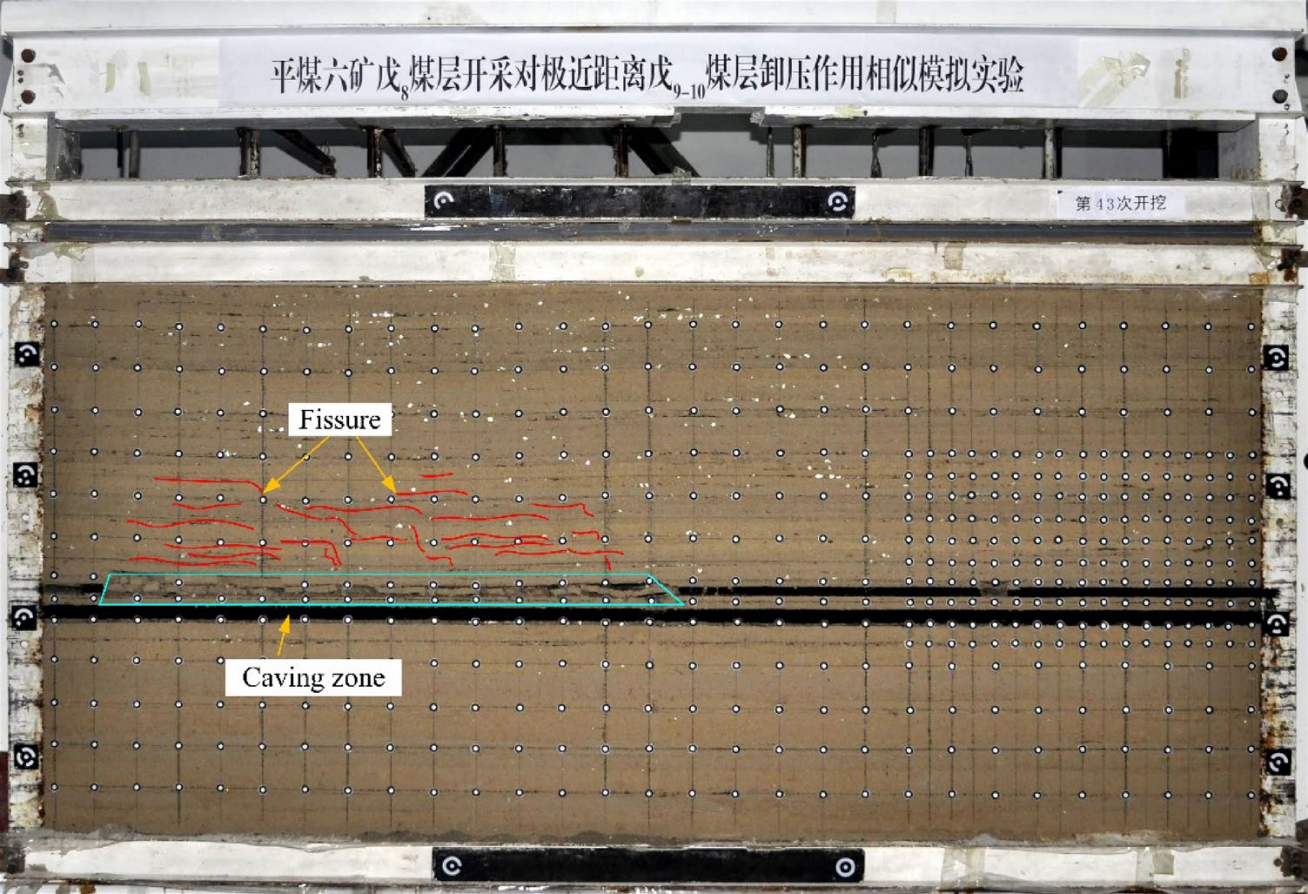
Mining advancement of 129 m.

**Fig. 13:**
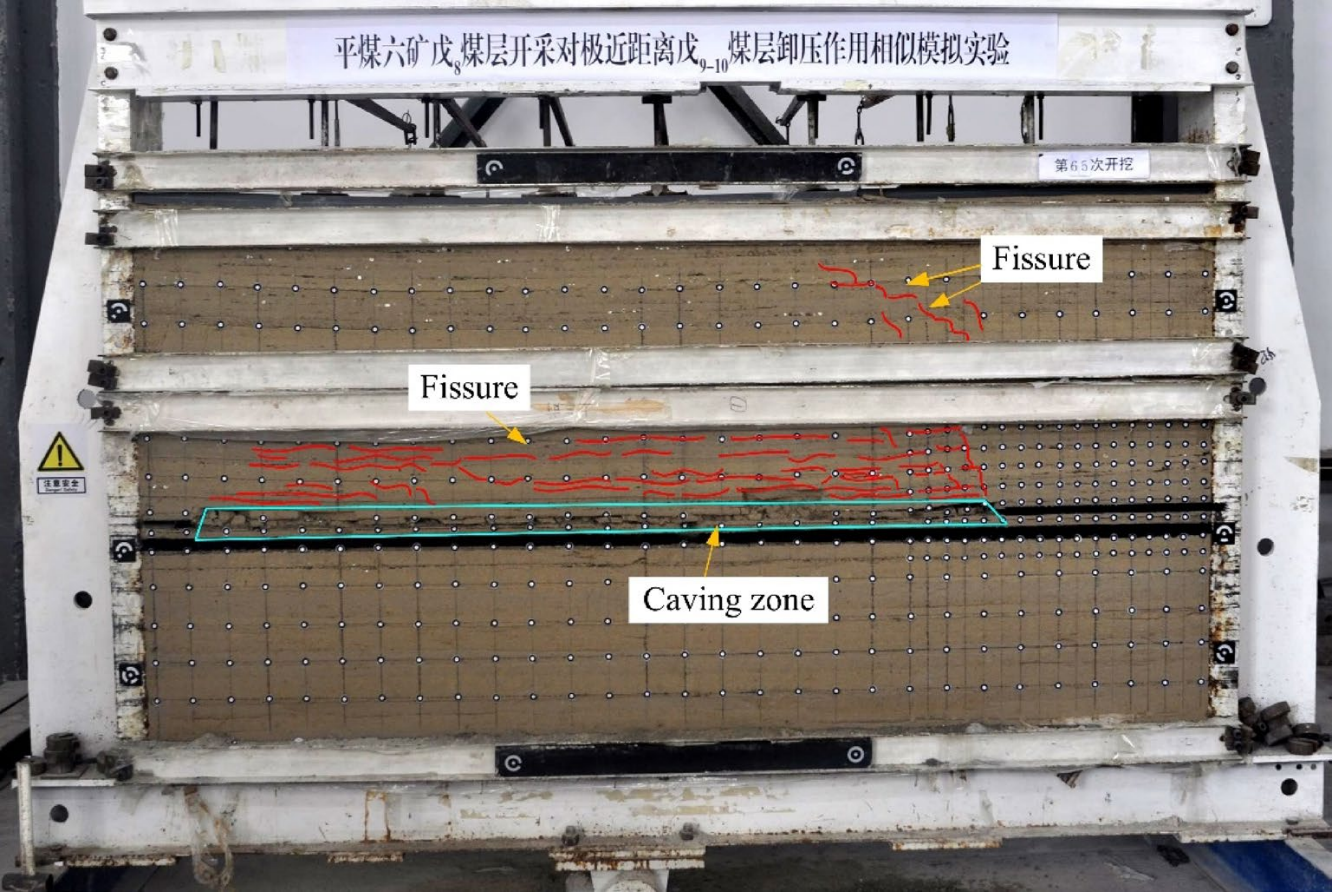
Mining advancement of 200 m.

**Fig. 14:**
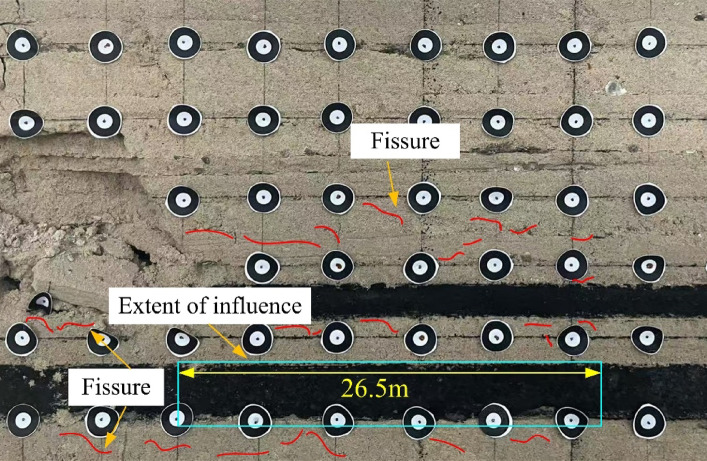
Final development of cracks in the underlying coal seam.

As shown in Figs. 10, 11, 12, 13 and 14, fractures in the rock strata are induced by mining activities. With the progression of excavation, the fractures continue to develop, and new fractures are formed. When the working face advances to 18 m, delamination begins to form in the immediate roof of the goaf, accompanied by gradual subsidence. Subsequently, horizontal fractures start to develop in the rock strata above the immediate roof and continue to propagate upward.

When the working face advances to 39 m, the roof experiences its initial collapse. The collapsing roof rock beam does not fall as a single intact block but fractures into multiple segments. The collapse height is approximately 2 m. The number of fractures above the roof increases and expands in the direction of the retreating face. The length of the fractures also increases, with a tendency to propagate further into the overlying strata.

When the working face advances to 129 m, marking the halfway point of extraction, the collapse range of the goaf roof continues to expand in the direction of face retreat. However, the collapse height remains relatively stable. The degree of fracture connectivity in the overlying strata intensifies, and the fractures exhibit significant propagation.

When the working face advances to 200 m, the coal seam is fully mined out. Two channel steels are added to the middle of the model to ensure stability and prevent forward tilting or collapse. At this point, the fractures in the goaf have propagated to the top of the model.

After the complete extraction, small fractures appear in the roof and floor of the coal seam. Although these fractures are not yet prominent, they signal the release of stress and indicate that the underlying coal seam in this area is undergoing an initial depressurization process. The fracture development extends approximately 26.5 m from the roadway rib of the overlying coal seam.

### Stress monitoring

#### Stress monitoring plan

During the construction of the test model, LY-350 micro pressure boxes were embedded at the specified monitoring points according to the preset plan. The strain signals from the micro-mechanical strain gauges inside the pressure boxes were used to monitor the stress on the floor of the underlying coal seam. The strain signals were received by the DH3816N static strain gauge, and the data were displayed and analyzed through the DHDAS signal acquisition and analysis system on the computer.

To study the stress variation pattern of the underlying coal seam at extremely close distances during the mining of the working face, a total of 8 stress monitoring points were set up. Each monitoring point uses an LY-350 micro pressure box to record the stress changes within 35 m to the right of the roadway rib. Starting from 35 m away from the right boundary, a pressure box is placed every 5 m, with a total of 8 pressure boxes arranged. The layout of the stress monitoring points for the similar material simulation test is shown in the figure above (Fig. 5).

#### Stress variation pattern

With the advancement of the working face, significant changes in stress were observed at each monitoring point. The main characteristics of these changes are as follows:

1. The vertical stress at monitoring points 1 to 4 in the underlying coal seam shows a trend of "increasing first, then decreasing" as the working face advances. This indicates that the monitoring points near the working face are initially affected by mining activities, leading to an increase in stress. Once the coal body fails, the stress in that area decreases and gradually shifts to deeper layers. The final vertical stresses at monitoring points 1 to 4 of the underlying coal seam are 0.44 MPa, 11.2 MPa, 17.32 MPa, and 18.33 MPa, respectively, all of which are lower than the pre-mining stress of 21.07 MPa, indicating they are within the depressurization zone.
2. The stress variation pattern at monitoring point 5 is similar to that at monitoring points 1 to 4, also showing a "first increasing, then decreasing" trend. However, the final vertical stress value at this point is higher than the original stress before mining, indicating that monitoring point 5 is not within the depressurization zone.
3. For the underlying coal seam monitoring points 6 to 8, located farther from the working face, the vertical stress gradually increases as the working face advances, but the rate of increase is relatively slow. This indicates that these points are affected by mining-induced stress, but the coal body has not failed and still maintains a high strength. The final stress values are 28.6 MPa, 29.41 MPa, and 33.6 MPa, all of which are higher than the pre-mining stress of 21.07 MPa, placing them within the plastic zone of ultimate equilibrium.

**Fig. 15:**
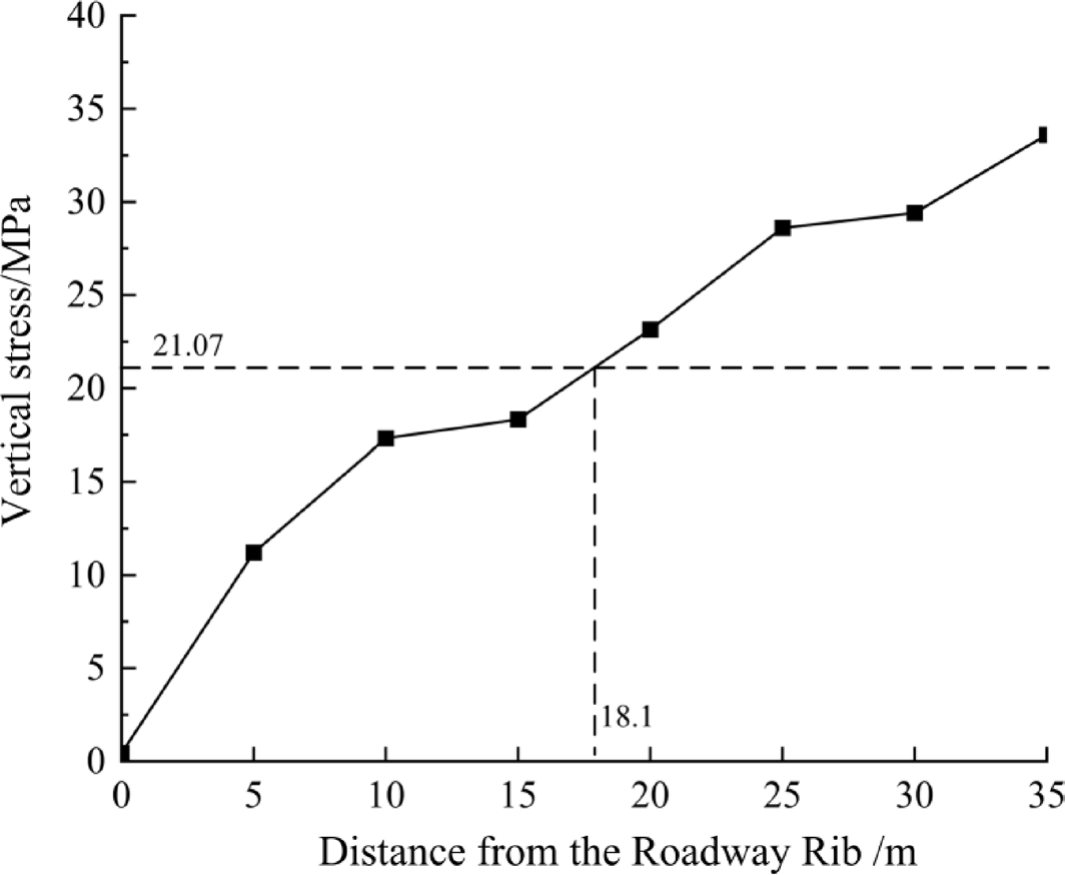
Variation curve of vertical stress in the underlying coal seam with distance from the tunnel wall.

Figure 15 shows the stress conditions at each monitoring point in the underlying coal seam after the completion of the working face mining. From this, it can be observed that:

1. The vertical stress within 0–18.1 m from the roadway rib of the working face is lower than the original stress, indicating that the area within 18.1 m from the roadway rib is a depressurization zone.
2. The vertical stress within the range of 18.1–35 m from the roadway rib of the working face is higher than the original stress, indicating that the area beyond 18.1 m from the roadway rib is a stress increase zone. At 35 m, there is still no observable trend of stress reduction. Based on the fundamentals of mine pressure theory, the range of 18.1–35 m can be classified as the plastic zone of ultimate equilibrium.

### Variation pattern of deformation coefficient in the underlying coal seam

During the depressurization process, the coal seam undergoes elastic and plastic deformation due to stress release, resulting in morphological changes and displace-ment of the coal body and surrounding rock strata. Usually, the "0.3%" deformation coefficient is used as the criterion for determining the pressure relief deformation of coal seam, which is intended to ensure that the deformation of coal seam is within a reasonable range.

**Fig. 16:**
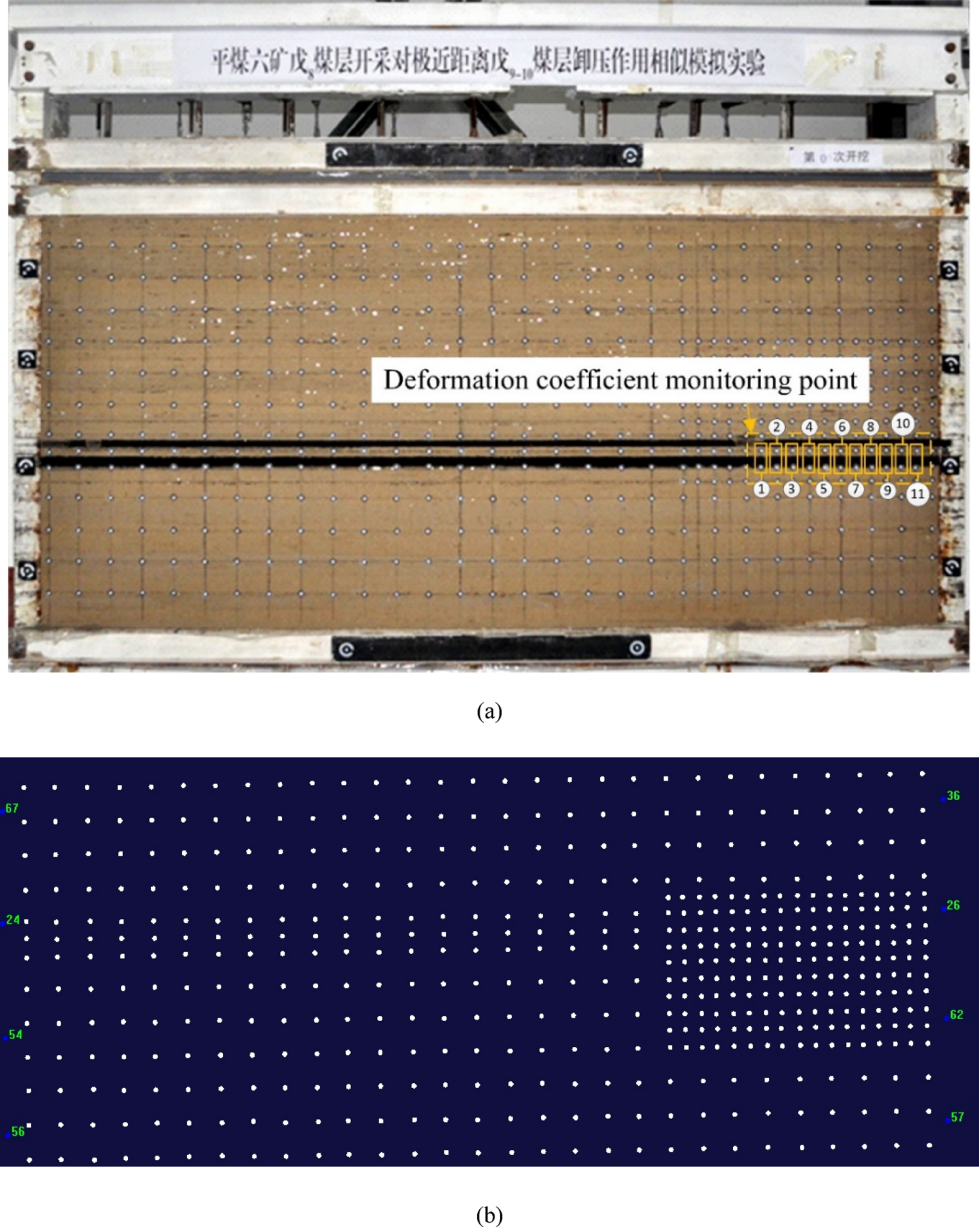
Deformation monitoring of coal and rock strata in the mining face based on basic digital speckle technology. (**a**) Arrangement of digital speckle monitoring points. (**b**) Identification of digital speckle monitoring points.

In this experiment, the XJTUDP 3D optical photogrammetry system was used to monitor displacement. Non-coded points with an inner diameter of 10 mm and an outer diameter of 20 mm were adhered to the model. The vertical spacing between adjacent non-coded points was 5 cm, with a horizontal spacing of 5 cm in dense areas and 10 cm in non-dense areas. A total of 11 groups of monitoring points were arranged, as illustrated in Fig. 16.

The range of the "depressurization desorption zone" is characterized by the deformation of the coal seam induced by depressurization. A deformation coefficient of 0.3% (3‰) is used as the threshold to determine the extent of the "depressurization desorption zone."

The determination of the range of the "depressurization desorption zone" is based on Eq. (3).

3 $${{\Delta \varepsilon } \mathord{\left/ {\vphantom {{\Delta \varepsilon } {(1 - \Delta \varepsilon_{0} )}}} \right. \kern-0pt} {(1 - \Delta \varepsilon_{0} )}} \ge 0.003$$

After depressurization, the coal seam undergoes volumetric expansion, which is typically expressed using the deformation coefficient:

4 $$k = \frac{V^{\prime} - V}{V} = \frac{V^{\prime}}{V} - 1$$

In the equation:* k* is the deformation coefficient, $V^{\prime }$ is the volume of the coal body after depressurization (in m^3^), $V$ is the volume of the coal body before depressurization (in m^3^). Before depressurization, the coal body primarily undergoes vertical compressive deformation. After depressurization, the coal body mainly expands vertically. Therefore, the deformation coefficient of the coal seam can be approximately calculated from vertical deformation, as shown in Eq. (5).

5 $$k \approx k_{c} = \frac{{h^{\prime}_{n \sim n + 1} }}{{h_{n \sim n + 1} }} - 1$$

The equation represents: ${k}_{c}$ is the vertical deformation coefficient of the coal body; $h_{n\sim n + 1}{\prime}$ is the distance between two adjacent points after pressure release, in meters (m); ${h}_{n\sim n+1}$ is the distance between two adjacent points before pressure release, in meters (m).

**Fig. 17:**
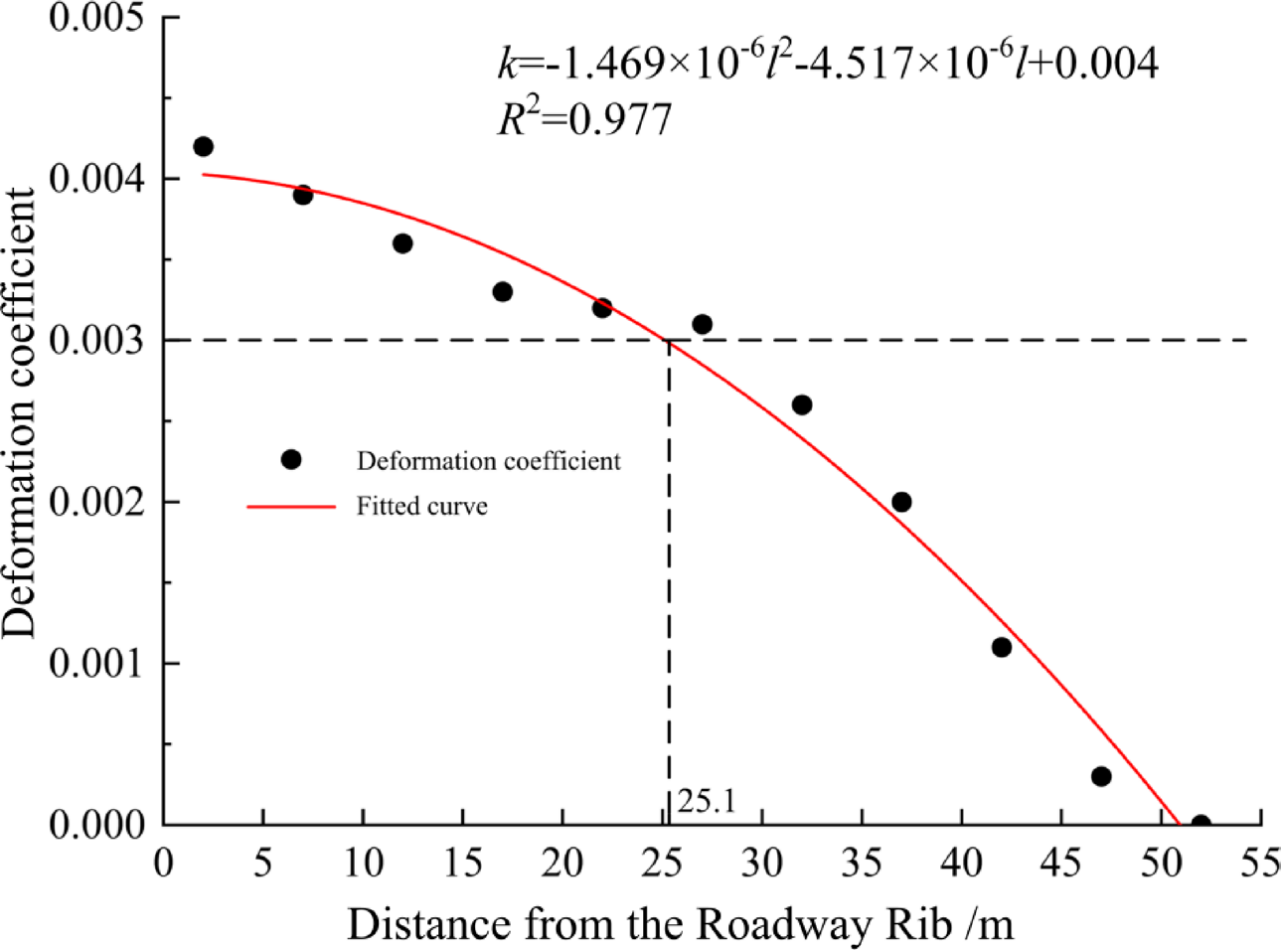
Fitting curve of deformation coefficient.

The calculation results from each monitoring point were fitted, as shown in Fig. 17. From the figure, it can be observed that the underlying coal seam undergoes expansion deformation due to mining disturbances from the overlying coal seam, with an impact range of up to 50 m. By substituting 3‰ into Eq. (6), the deformation coefficient of the underlying coal seam is calculated to be 3‰, which corresponds to a distance of 25.1 m from the working face rib. This indicates that the influence of the deformation coefficient is significant within a distance of 25.1 m from the working face rib.

6 $$k = - 1.469 \times 10^{ - 6} l^{2} - 4.517 \times 10^{ - 6} l + 0.004$$

## On-site monitoring of gas pressure and gas content

### Underground on-site testing plan

In the Wu _8_-32030 high-level roadway, a total of five measurement points were arranged. The locations of the gas pressure and gas content measurement points were identical, allowing for simultaneous measurement of both parameters within the same borehole. This "one borehole, dual-purpose" setup enhances measurement efficiency.

The specific locations of the measurement points relative to the cut are as follows:

1. Measurement Point 1: 365 m from the cut
2. Measurement Point 2: 300 m from the cut
3. Measurement Point 3: 235 m from the cut
4. Measurement Point 4: 160 m from the cut
5. Measurement Point 5: 85 m from the cut

Each measurement point was equipped with five boreholes:

1. Measurement Point 1: Boreholes 1–1, 1–2, 1–3, 1–4, and 1–5
2. Measurement Point 2: Boreholes 2–1, 2–2, 2–3, 2–4, and 2–5
3. Measurement Point 3: Boreholes 3–1, 3–2, 3–3, 3–4, and 3–5
4. Measurement Point 4: Boreholes 4–1, 4–2, 4–3, 4–4, and 4–5
5. Measurement Point 5: Boreholes 5–1, 5–2, 5–3, 5–4, and 5–5

**Fig. 18:**
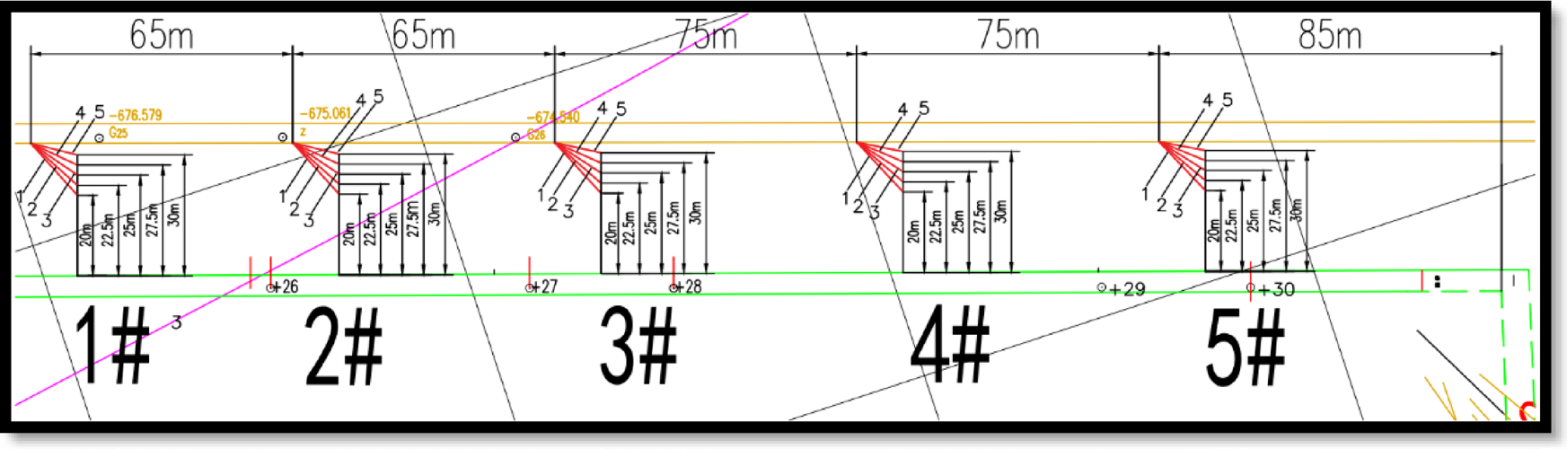
Plan view of borehole layout for gas content and gas pressure.

For detailed parameters, please refer to Tables 3, 4, 5, 6, 7 and Fig. 18.

**Table 3:** Borehole parameters of measurement point 1.

Borehole number	Horizontal angle (°)	Distance between borehole endpoint and rib of the machine roadway (m)	Vertical inclination angle (°)	Borehole depth (m)
1–1	48°	20	72°	39.3
1–2	42°	22.5	72°	38.6
1–3	35°	25	72°	38
1–4	25°	27.5	72°	37.5
1–5	14°	30	72°	37.3

**Table 4:** Borehole parameters of measurement point 2.

Borehole number	Horizontal angle (°)	Distance between borehole endpoint and rib of the machine roadway (m)	Vertical inclination angle (°)	Borehole depth (m)
2–1	48°	20	72°	38.6
2–2	42°	22.5	72°	37.9
2–3	34°	25	72°	37.3
2–4	25°	27.5	72°	36.8
2–5	13°	30	72°	36.6

**Table 5:** Borehole parameters of measurement point 3.

Borehole number	Horizontal angle (°)	Distance between borehole endpoint and rib of the machine roadway (m)	Vertical inclination angle (°)	Borehole depth (m)
3–1	48°	20	71°	37
3–2	41°	22.5	71°	36.3
3–3	33°	25	71°	35.7
3–4	24°	27.5	71°	35.2
3–5	13°	30	71°	35

**Table 6:** Borehole parameters of measurement point 4.

Borehole number	Horizontal angle (°)	Distance between borehole endpoint and rib of the machine roadway (m)	Vertical inclination angle (°)	Borehole depth (m)
4–1	47°	20	71°	37.1
4–2	41°	22.5	71°	36.3
4–3	33°	25	71°	35.7
4–4	23°	27.5	71°	35.3
4–5	12°	30	71°	35

**Table 7:** Borehole parameters of measurement point 5.

Borehole number	Horizontal angle (°)	Distance between borehole endpoint and rib of the machine roadway (m)	Vertical inclination angle (°)	Borehole depth (m)
5–1	47°	20	71°	37.1
5–2	40°	22.5	71°	36.3
5–3	32°	25	71°	35.8
5–4	22°	27.5	71°	35.3
5–5	11°	30	71°	35.1

### Analysis of gas content test results

**Fig. 19:**
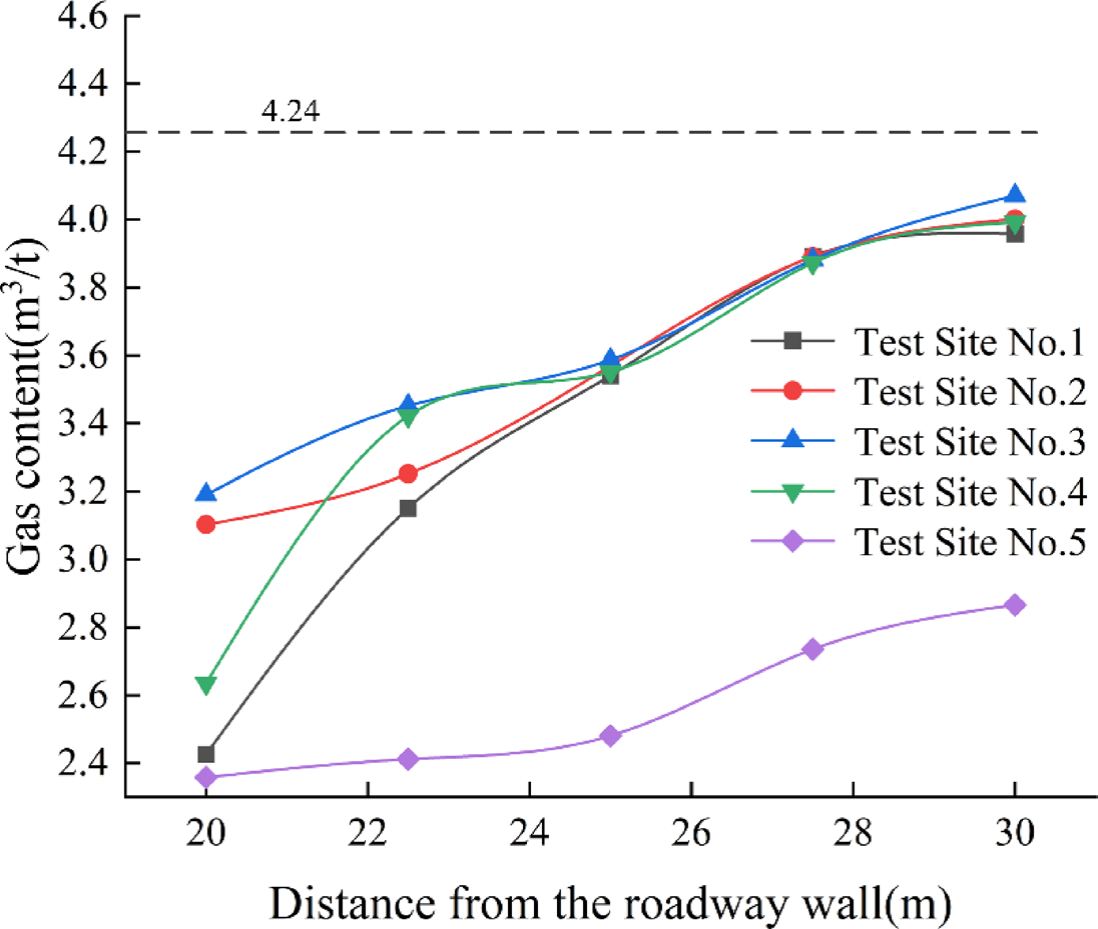
Gas content characteristics at measurement points in the ultra-close underlying coal seam after face mining.

The gas content of the coal samples was measured through gas desorption tests conducted both underground and in the laboratory, and the results were plotted as a curve. The original gas content was determined to be 4.24 m^3^/t. See Fig. 19 for details.

After the working face was mined, the gas content at measurement points 1, 2, 3, 4, and 5 in the underlying coal seam within 20–30 m from the roadway side was lower than the original gas content. This indicates that gas desorption occurred to some extent, leading to a reduction in gas content in the ultra-close underlying coal seam after the working face was mined. Among them, measurement point 5 showed the greatest decrease in gas content, while the gas content trends at measurement points 1 to 4 were similar. At a distance of 30 m from the roadway side, the gas content at two measurement points approached the original level. Based on the observed range in the underground field measurements, the gas influence range from the roadway side was determined to be approximately 29 m.

### The analysis of gas pressure test results

**Fig. 20:**
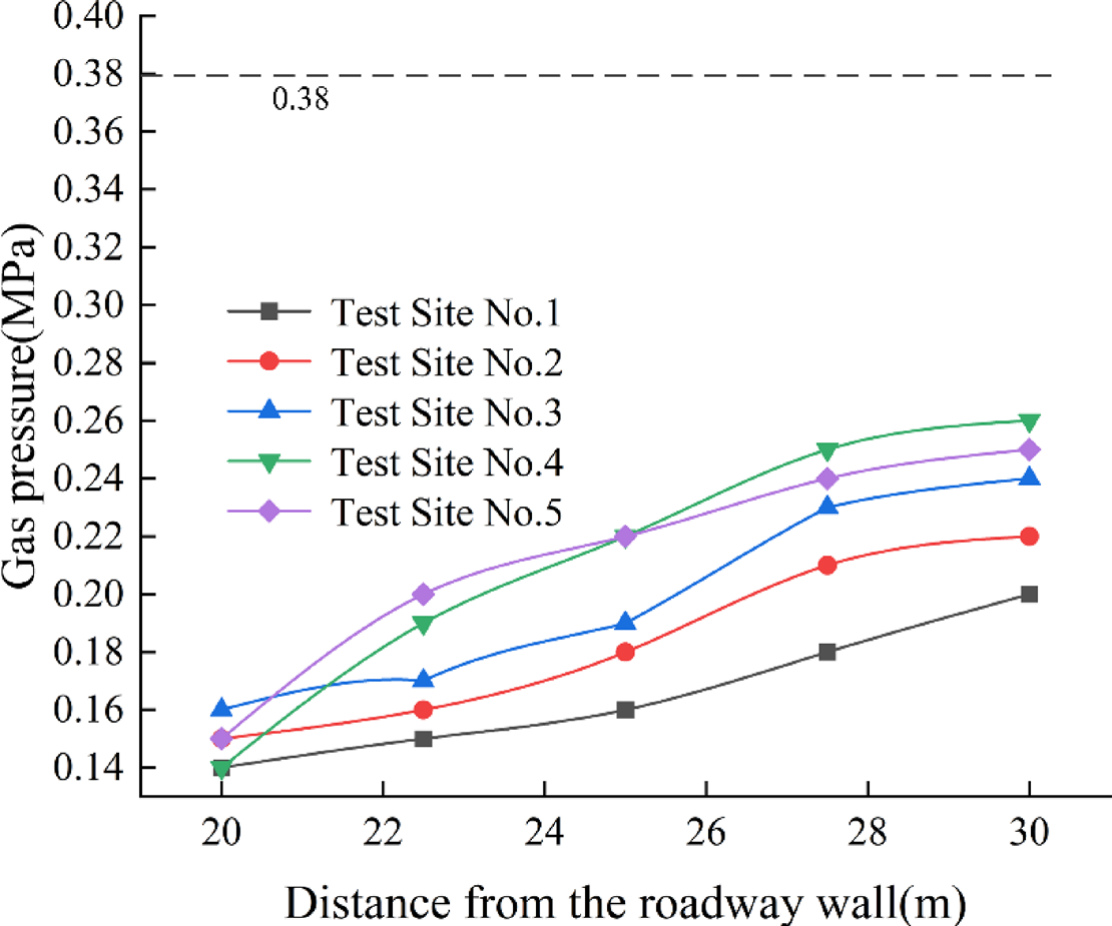
Gas pressure characteristics at measurement points in the ultra-close underlying coal seam after face mining.

The final gas pressure values were obtained using a long-term passive gas pressure measurement method and plotted as a curve. The original gas pressure was measured at 0.38 MPa. See Fig. 20 for details.

As shown in Fig. 20, after the working face mining, the gas pressure at monitoring points 1, 2, 3, 4, and 5 in the ultra-close underlying coal seam increases with the distance from the roadway side, which is consistent with the overall trend of gas content changes. Among them, at monitoring points 2 to 5, the gas pressure increases rapidly within the range of 20–27.5 m, while the growth rate of gas pressure significantly slows down in the range of 27.5–30 m. At monitoring point 1, the rate of increase remains consistently high from 20 to 30 m. Through comprehensive analysis of monitoring points 1 to 5, it can be determined that the destressed range of the underlying coal seam is 27.5 m.

Based on a comprehensive analysis, it can be concluded that under the influence of mining activity, the gas pressure within 27.5 m from the roadway side in the ultra-close underlying coal seam decreases significantly.

In summary, the on-site gas testing results confirmed the final fracture development extent and the influence range of deformation coefficients observed in the previous physical similarity simulation experiments. Both gas content and gas pressure exhibited similar trends, increasing with the distance from the working face rib, yet remaining lower than the original gas testing results. This indicates that under the influence of overlying coal seam mining, gas desensitization can be effectively carried out within this range.

## Conclusion

1. Through numerical simulation analysis, it was found that after the mining of the overlying coal seam, a certain range of stress relief and plastic zones developed within the ultra-close underlying coal seam. As the mining operation progressed, these zones gradually expanded and eventually stabilized, with a final stable influence range of approximately 25 m.
2. Through physical similarity simulation experiments of the ultra-close coal seam, it was determined that after the overlying coal seam was mined, the vertical stress relief range in the ultra-close underlying seam reached 18.1 m, and the fracture propagation influence range extended to 26.5 m. Displacement monitoring of the underlying seam showed that the deformation coefficient of the coal seam decreased with increasing distance, and a polynomial relationship between the deformation coefficient and the distance from the roadway was established. The influence range was approximately 25.1 m.
3. Based on field monitoring of gas pressure and gas content, it was found that under the disturbance caused by the overlying coal seam mining, the gas pressure relief and outburst prevention influence range in the ultra-close underlying coal seam was about 27.5 m. These monitoring results primarily reflected the actual outburst prevention effect in the ultra-close coal seam.
4. By integrating the results of numerical simulation, physical similarity simulation, and field measurements, the final determined gas pressure relief and outburst prevention range of the ultra-close coal seam was approximately 27.5 m. Considering that field monitoring results directly reflect the actual outburst prevention effect, and that the ultimate goal of the study is focused on defining the outburst prevention range, priority was given to the field monitoring results, with numerical simulation and physical similarity simulation serving as supplementary methods. Consequently, the pressure relief and outburst prevention range was determined. Operating within this range can effectively reduce the risk of coal and gas outbursts, providing an important reference for the rational layout of outburst prevention measures and the planning of subsequent mining roadways, thereby possessing significant engineering application value.

## Data Availability

The datasets used and/or analysed during the current study available from the corresponding author on reasonable request.
